# Minimally Invasive Repair Options for Symptomatic Larrey Hernia in an Adult: A Case Report and Literature Review

**DOI:** 10.7759/cureus.93742

**Published:** 2025-10-02

**Authors:** Wilson S Peñafiel-Pallares, Scott J Roth

**Affiliations:** 1 General Practice, Universidad de las Américas, Quito, ECU; 2 General Surgery, University of Kentucky College of Medicine, Lexington, USA

**Keywords:** laparoscopic surgeries, morgagni-larrey hernia, polypropylene mesh, surgical case reports, symptomatic hernia

## Abstract

Morgagni-Larrey hernias (MLH) are rare congenital anterior diaphragmatic defects, and Larrey hernias are a less common variant that typically occurs on the left side. Their rarity and often asymptomatic nature in adults make diagnosis and treatment challenging. A 44-year-old woman experienced intermittent left upper quadrant abdominal pain for one year, with associated nausea and vomiting. Her past medical history included a laparoscopic cholecystectomy and a right inguinal hernia repair. Imaging confirmed a left-sided anterior diaphragmatic defect consistent with a Larrey hernia. Laparoscopic surgery revealed herniation of omentum and colon, which was repaired with primary suture closure and polypropylene mesh reinforcement. The patient remained asymptomatic, with radiographic confirmation of repair and no recurrence at five months. This case highlights the importance of considering MLH in adults with nonspecific gastrointestinal symptoms. Early diagnosis and surgical intervention are key to preventing complications such as bowel obstruction and respiratory compromise.

## Introduction

Morgagni-Larrey hernias (MLH) are rare congenital defects involving the anterior, parasternal portion of the diaphragm. They are classically divided into the following three categories based on location: Morgagni hernias (right-sided), Larrey hernias (left-sided), and bilateral hernias [1]. MLH results from the failure of the pars tendinalis to fuse with the pars sternalis during embryonic development [2]. Other types of congenital diaphragmatic hernia include Bochdalek, central, and congenital hiatal hernias [1].

Although acquired cases have also been described in association with trauma, previous thoracoabdominal surgery, or conditions that chronically increase intra-abdominal pressure, the vast majority of MLH are congenital in origin [3,4]. MLH account for approximately 2-4% of all congenital diaphragmatic hernias, with Morgagni defects being by far the most common and Larrey hernias the least frequent [1]. The clinical presentation varies and is largely dependent on the hernia’s contents. Large retrospective series have reported dyspnea and chest pain as the most common presentations, followed by gastrointestinal complaints [1,5]. Small case series have reported a mean age of presentation of around 30 years. Broader reviews suggest that the average age at diagnosis in adulthood is approximately 50 years, often incidental [3,4].

## Case presentation

**Figure 1:**
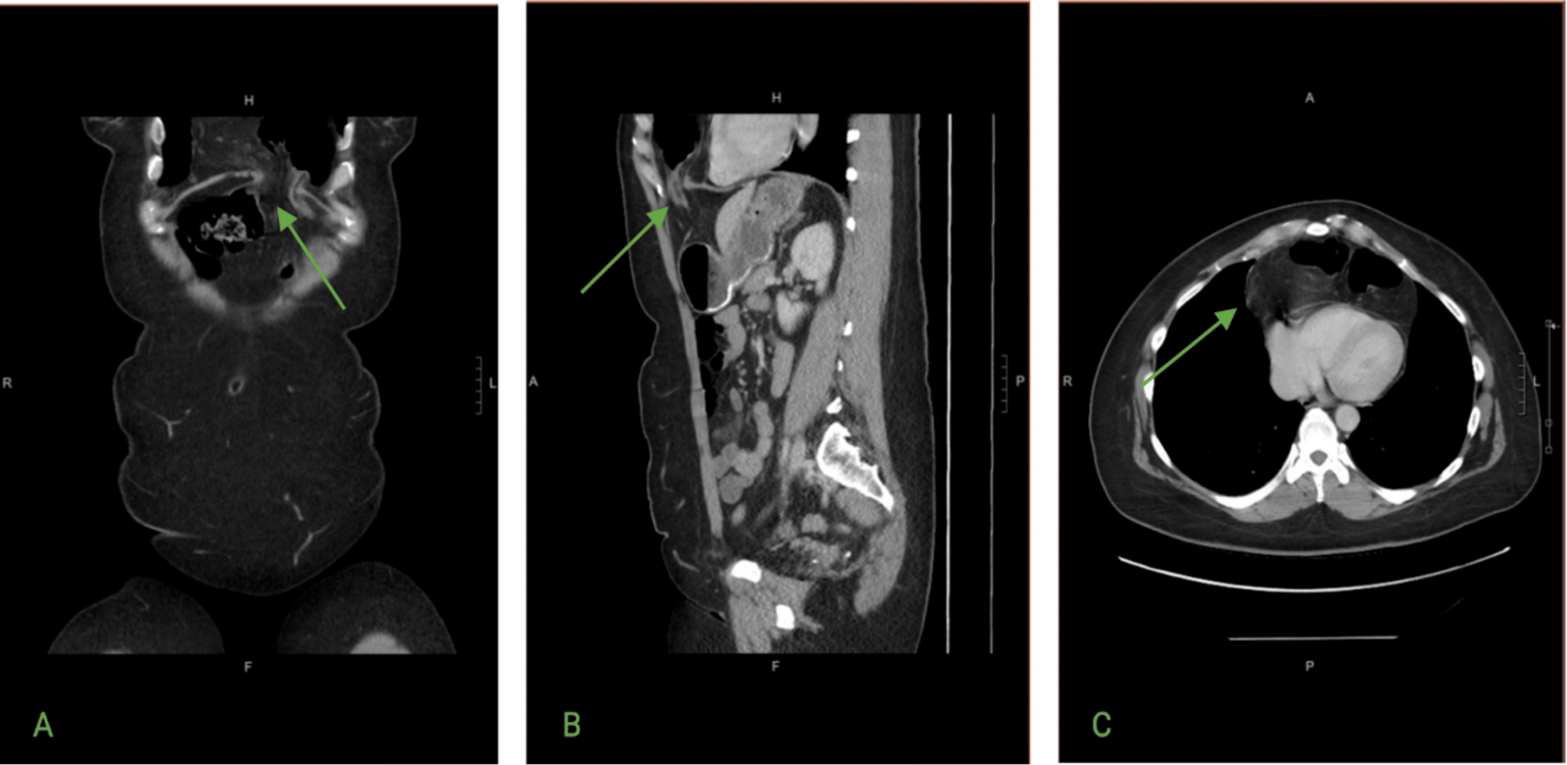
Computed tomography imaging of a Larrey hernia. (A) Coronal CT view showing a left-sided anterior diaphragmatic defect with herniation of abdominal contents into the thoracic cavity (green arrow). (B) Sagittal CT view demonstrating the herniated bowel loops passing through the diaphragmatic defect into the left thoracic cavity (green arrow). No evidence of significant mediastinal shift or compression of adjacent thoracic structures is observed. (C) Axial CT image showing a left-sided anterior diaphragmatic defect with herniation of abdominal contents (green arrow). The defect is consistent with a Larrey hernia.

**Figure 2:**
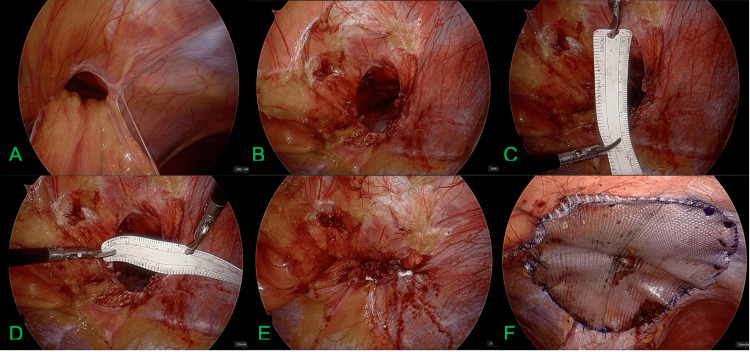
Intraoperative view of Larrey hernia repair. (A) Initial laparoscopic view showing the diaphragmatic defect adjacent to the xiphoid process with herniation of omentum and transverse colon into the thoracic cavity. (B-D) Hernia defect measuring approximately 3x4 cm. (E) Primary closure of the diaphragmatic defect using nonabsorbable sutures. (F) Placement of a polypropylene mesh for reinforcement, ensuring tension-free repair.

We report a case of a 44-year-old woman with a one-year history of intermittent left upper quadrant abdominal pain without changes in bowel habits. Each episode lasted 2-3 hours, resolved spontaneously, and was associated with nausea and vomiting. Past medical history was significant for a laparoscopic cholecystectomy and a right inguinal hernia repair in 1985. Computed tomography (CT) revealed a left-sided anterior diaphragmatic defect with herniation of abdominal contents into the thoracic cavity. The herniated structures included bowel loops located superior to the diaphragm (Figures 1A-1C, 2A-2F). No significant mediastinal shift or compression of adjacent thoracic structures was observed.

**Figure 3:**
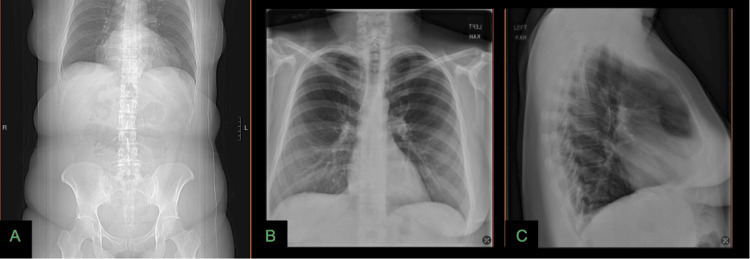
Preoperative and postoperative radiographic imaging of a Larrey hernia. (A) Preoperative CT scout image demonstrating an opacity overlying the mediastinum with soft tissue and air consistent with herniation of a hollow viscus. (B-C) Anteroposterior and lateral chest X-rays obtained five months after Larrey hernia repair, showing normal contour of the hemidiaphragm; no intrathoracic bowel loops; absence of retrosternal masses; clear lung fields; and no pleural effusion.

The hernia, containing omentum and colon, was successfully repaired in November 2024 by laparoscopic primary suture closure with polypropylene mesh reinforcement (Figures 2A-2F). At the five-month follow-up, the patient remained asymptomatic. A chest X-ray confirmed appropriate closure of the diaphragmatic defect (Figures 3A-3C). This case illustrates the management of a Larrey hernia using laparoscopic techniques and mesh repair, emphasizing the importance of considering this diagnosis in adults with nonspecific gastrointestinal symptoms. The patient provided informed consent for publication and expressed satisfaction with the outcome.

## Discussion

Morgagni-Larrey hernias (MLH) are the most frequent anterior diaphragmatic congenital defects. The true incidence in the general population remains uncertain, largely because they are often asymptomatic and may go undetected for decades. Among anterior diaphragmatic hernias, Morgagni defects predominate, accounting for approximately 90% of cases, bilateral defects for about 8% of cases, and Larrey hernias for only 2% of cases [1]. When a diagnosis occurs in adulthood, it is often incidental during imaging for unrelated conditions. Reported mean ages vary, from 29.6 years in a younger surgical cohort described by Abraham et al. to 53 years in a case series by Bennani et al., which also noted a slight female predominance [3-6]. Larger studies are still needed to establish the true epidemiology of this entity.

Clinical manifestations depend on the hernia’s contents. Typical findings include herniation of the transverse colon, omentum, stomach, and, less commonly, the liver [1,5]. While dyspnea and chest pain are the most frequently reported symptoms, gastrointestinal complaints may also occur. Our patient presented with intermittent abdominal pain, nausea, and vomiting; these are less common features but have been documented in prior reports. If left untreated, these hernias may lead to serious complications such as bowel obstruction, strangulation, perforation, or respiratory compromise due to compression of thoracic structures [5].

Imaging is essential for diagnosis. Plain radiography may show a retrosternal opacity or abnormal diaphragmatic contour (Figure 3). CT remains the modality of choice, offering precise localization of the defect and characterization of the hernia contents [1,3,6]. In our case, CT confirmed herniation of omentum and colon without mediastinal shift, guiding surgical planning.

Due to its rarity, specific surgical guidelines for MLH repair have not been established. Nonetheless, surgical repair is recommended in both symptomatic and asymptomatic patients due to the risk of life-threatening complications [1]. Approaches include transabdominal or transthoracic access. The transthoracic route allows easier dissection of the sac from the mediastinum and pleura, but is limited by single-lung ventilation and reduced visualization of abdominal contents [6]. The transabdominal approach, particularly via laparoscopy, offers excellent exposure of both thoracic and abdominal structures [1,6].

Techniques include primary suture closure, suture with mesh reinforcement, or mesh placement alone. Mesh reinforcement is recommended in adults with large defects or when tension-free closure is not feasible, as it appears to reduce the recurrence risk [2,4,7]. Although pediatric data suggest benefits of patch use, in adults, laparoscopic mesh-reinforced closure is widely adopted [8]. In our patient, laparoscopic primary closure with polypropylene mesh reinforcement was effective, with no recurrence at five months. Although our patient remained asymptomatic during this period, it is important to note that recurrences may occur later. Oppelt et al. emphasized that an optimal follow-up period of at least 12-24 months is advisable to adequately assess for recurrence after surgical repair of Morgagni-Larrey hernias [1].

Minimally invasive techniques have become increasingly popular for MLH repair. Laparoscopic repair offers several advantages, such as shorter hospital stays, faster recovery, better cosmetic results, and improved visualization of the hernia contents [6,9]. Reports by Horton et al. and Bennani et al. have highlighted the safety and effectiveness of laparoscopic repair in adult patients, including Larrey hernias [4,9]. Similarly, Palma et al. described mesh-reinforced laparoscopic repair in combination with bariatric procedures, emphasizing its adaptability [6,8]. This case adds to the growing body of evidence supporting laparoscopic repair with mesh reinforcement as a safe and effective treatment option.

The primary limitation of this report is its short follow-up period, which does not permit conclusions regarding long-term recurrence. Nonetheless, the patient remains asymptomatic with radiographic confirmation of repair.

## Conclusions

Larrey hernia, a variant of Morgagni hernia, represents a diagnostic and therapeutic challenge due to its infrequent occurrence in adults and often nonspecific clinical presentation. This case highlights the crucial role of computed tomography in establishing an accurate diagnosis and guiding surgical planning. Minimally invasive laparoscopic repair with mesh reinforcement provides a safe, effective, and durable treatment, minimizing postoperative morbidity and reducing the risk of recurrence, as demonstrated in the present case. The growing body of evidence, combined with our experience, supports laparoscopy as the preferred approach for adult Larrey hernias when mesh reinforcement is feasible. However, individualized surgical planning remains essential, considering defect size, comorbidities, and intraoperative findings.
